# Issues in defining/extracting collocations in Japanese and Korean: Empirical implications for building a collocation database

**DOI:** 10.1016/j.heliyon.2016.e00189

**Published:** 2016-11-29

**Authors:** Jong-Seung Park, Tohru Seraku, Jieun Kiaer

**Affiliations:** a Department of Japanology, Gangneung-Wonju National University, 7 Jukheon-gil, Gangneung-si, Gangwon-do 210-702, South Korea; b Department of Japanese Interpretation and Translation, Hankuk University of Foreign Studies, South Korea; c Faculty of Oriental Studies, University of Oxford, United Kingdom

**Keywords:** Linguistics

## Abstract

Collocations in Japanese and Korean have been studied extensively based on statistical tools. The criteria for collocations in these languages, however, have not been fully established in the literature, and it is not obvious whether all statistically significant combinations of words could be regarded as collocations. In this article, we point out empirical problems in extracting collocations in Japanese and Korean, and provide a standard for identifying collocations (to be separated from “free combination” and “idiom”) in these languages. We concentrate on the discussion of empirical aspects of collocation research, rather than the statistical analyses of collocational patterns. As a preliminary to developing a database for Japanese-Korean contrastive work on collocations, the present study focuses on ten “Chinese-words” (漢語). We show that (i) the co-occurring verbs for eight Chinese-words in Korean all carry over to Japanese (but not vice versa); (ii) in the other two cases, Korean exhibits co-occurring verbs not found in Japanese; (iii) language-specific patterns of verb co-occurring are also observed in some instances. Overall, it is indicated that a much wider variety of co-occurring verbs are found in Japanese than in Korean.

## 1 Introduction

Collocation has been widely utilised in language teaching; it refers to related phrases or clauses which co-occur in a statistically significant way (Strafella and Maekawa, 2015). For instance, according to *The Oxford Collocations Dictionary for Students of English* (2nd edition, 2009), the noun *influence* frequently co-occurs with the verbs in (1):

(1) *have*, *gain*, *exercise*, *exert*, *retain*, *lose*, *expand*, *extend*, *increase*, *spread*

With the development of corpus linguistics, there has been a growing body of work which makes use of statistical tools to extract collocations from corpora. The data thus collected, however, include “free combinations” and “idioms,” which raises the issues of (i) whether these are to be regarded as collocations and (ii) if not, what criteria are to be set out to constrain the range of collocations. These issues, in our view, have not been seriously addressed, especially for Japanese and Korean. For instance, as will be pointed out in Section 3.6, not all statistically significant combinations of words are not regarded as collocations (at least, with respect to the standard assumed in the present article). Therefore, whilst statistical analyses constitute important approaches to the study of collocations, empirical issues involved in the extraction of collocations are in need of thorough discussion and the standard for collocations must be established which could supplement such statistical approaches.

In this article, we aim to provide an empirical foundation to construct a collocation database to be employed for contrastive analyses of Japanese and Korean. Thus, rather than presenting a rigid statistical account, we focus on (i) the discussion of empirical issues in detecting collocational units in these languages and (ii) the presentation of a case study for a specific type of syntagmatic sequence which involves “Chinese-word” (kango, 漢語), a type of expression observed in both Japanese and Korean.

More specifically, we will provide the standard for collocations by applying Im’s (2006) criteria for Korean collocations to Japanese data. This standard will serve as a ground on which we analyse the syntagmatic sequence “Chinese-word + case particle + verb.” Through this case study, it will be revealed that, in spite of notable cross-language differences, there is a strong tendency that a much wider variety of verbs co-occur with Chinese-words in Japanese than in Korean. For data collection, we used the following two corpora and concordancer:

• IntelliText 2.6 (The University of Leeds, 2011), a 250 million-word corpus of written Japanese
• Sejong Corpus (The National Institute of Korean Language, 2010), a 200 million-word corpus of written Korean
• Kkokkoma Korean Analyzer (Seoul National University, 2009)

The organisation of this article is as follows: Section 2 first surveys the notion of collocation and the issues in extracting collocations from corpora. Section 3 then offers the standard for identifying collocations in Japanese and Korean based on Im’s (2006) proposal. With respect to this standard, Section 4 reports a case study of collocational units involving “Chinese-words.” Finally, Section 5 summarises our main findings and points out some future directions.

## 2 Background

### 2.1 Definition

“Collocation” has been variously defined in the literature (Cowie, 1994: 3169; Firth, 1968: 182; Hong, 1995: 425; Kim, 2000; Wanner, 1996). There are two major views on the concept of collocation (Im, 2006: 147):

(2) “Lexeme restriction”-based definition

Hausmann (1984), Hong (1995), Im (2002), Kim (2000), Lee, 1998, Lee, 2004, Manning and Schütze (1999), Mel’čuk, (1998)

(3) “Combination frequency”-based definition

Carter (1987), Cruse (1986), Halliday (1966), Halliday and Hasan (1976), Hong et al. (2001), Kang (1998), Sinclair, 1966, Sinclair, 1991, Smadja (1993)

In (2), a combination restricted by the selectional restrictions imposed on each lexeme is recognised as a collocation. By contrast, in (3), a combination of two lexemes whose syntagmatic co-occurrence is frequent (against a threshold) is viewed as a collocation. According to (3), an instance of “free combination” may be treated as a collocation if it is frequently attested, and pairs of lexemes with a low frequency are largely excluded. Given our research purpose stated in Section 1, we construe collocation in line with the “lexeme restriction”-based approach (2).

From the perspective of this approach, a collocation refers to a polar binary relation of lexical dependence, where a dominating lexical unit α (called “base”) selects a dominated lexical unit β (called “collocate”). In a collocation “noun + verb” in Japanese and Korea, a noun is a “base,” while a verb is a “collocate” (Im and Kim, 2002: 289). In the Japanese sequence *kusuri-o nomu* ‘take a medicine’ (4), the noun *kusuri* ‘medicine’ serves as a base in this collocational relation, and it selects the verb *nomu* ‘drink’ as a collocate. (Japanese examples are romanised in the Kunrei style. The glosses used in this article are as follows: ACC = accusative case particle, DECL = declarative clause marker, NOM = nominative case particle, PAST = past tense marker, TOP = topic particle.)

**Table:** 

(4)	*kusuri-o*	*nomu*	[Japanese]
	medicine-ACC	drink	
	‘take a medicine’		

According to the Japanese way of teaching Japanese language, collocations are often seen as a fixed association of words, a unit comparable to “rengo-teki kanyôku” (associated idiom) (Miyaji, 1985) and “rengo” (associated words) (Kunihiro, 1997). From the viewpoint of language education, it may be reasonable to conceive of free combinations as collocations in order to, e.g., prevent language transfers (Ooso, 2005). For instance, ‘take a medicine’ is expressed as (5) in Korean, where *mekta* is used. The Korean learners of Japanese may thus produce the ungrammatical phrase (6), where *taberu* is disallowed (cf. (4)). (Korean examples are romanised in the Yale style.)

**Table:** 

(5)	*yak-ul*	*mekta*	[Korean]
	medicine-ACC	eat	
	‘take a medicine’

**Table:** 

(6)	*kusuri-o	*taberu*	[Japanese]
	medicine-ACC	eat	
	Int. ‘take a medicine’		

Since our main objective is to provide a foundation for building a database for linguistic purposes (rather than for educational purposes), we do not treat free combinations as collocations in our research.

Turning to the issue of identifying collocations from corpora, it is essential to clarify which statistical indicators are used. In the present work, we will adopt three statistical indicators. These will be explicated in turn below.

Firstly, the “*t*-score” is utilised to compare the frequency of a base with that of a collocate with respect to the total number of words in a corpus. The *t*-score is suitable for evaluating collocations highly used in speech and discourse.

Secondly, the “*MI*-score” indicates to what extent a base encodes information concerning a collocate (Oakes, 1998: 63–65). The *MI*-score might not be reliable when the corpus size is not large as it may overemphasise expressions with a low frequency (Mikuni and Komori, 2008: 60). In this respect, our Japanese and Korean corpora, each of which encompasses more than 200 million words, are reasonably large in size.

Thirdly, the “Dice Coefficient” is adopted to measure the strength of a collocation solely based on the frequency relation between a base and a collocate. Chujo and Uchiyama (2004) report that the Dice Coefficient, although the calculation is simple, is a useful measure to extract “genre-specific words” (see also Ishikawa (2008)).

Our analysis makes use of these three indicators, taking into consideration (i) the co-occurrence frequency, (ii) the frequency of a base, (iii) the frequency of a collocate, and (iv) the total number of words in a corpus. In (7), *fA* stands for the frequency of a base *A*, *fB* the frequency of a collocate *B*, and *w* the total number of words in a corpus.

**Table:** 

(7)	Dice Coefficient, *MI*-score, *t*-score
	$\begin{array}{ccc} D=2\times \frac{fAB}{fA+fB} & I=\log2\frac{fAB\times W}{fA\times fB} & T=\frac{\left(fAB-\frac{fA\times fB}{W}\right)}{\sqrt{fAB}} \end{array}$

### 2.2 Issues in Identifying Collocations

Table 1 presents various statistical information relating to the association between the verb *yomu* ‘read’ as a base and its co-occurring lexeme in Japanese. Table 1 uncovers several difficulties in identifying collocations, as we will point out below.

Firstly, it might appear that the verb *yomu* ‘read’ selects a noun such as *hon* ‘book’ and *kiji* ‘article.’ In fact, it is widely assumed in the syntax literature that (i) a verb is a head of Verb Phrase and (ii) if a verb is transitive, it selects an object NP as an (internal) argument (e.g. Carnie (2012)). Here, an argument is assumed to be an NP (not a noun); this is because in (8), what *yomu* selects is not the noun *hon* ‘book’ but the whole part of the NP *omosiroi hon* ‘interesting book.’

**Table:** 

(8)	*omosiroi*	*hon-o*	*yonda*	[Japanese]
	interesting	book-ACC	read.PAST	
	‘I read an interesting book.’			

In the case of collocation, however, a noun selects a verb. Consider (9).

**Table:** 

(9)	*hon-o*	*yukkurito*	*yonda*	[Japanese]
	book-ACC	slowly	read.PAST	
	‘I read a book slowly.’			

In (9), the noun *hon* ‘book’ is in a collocational relation to the verb *yonda* ‘read’ (not the VP *yukkurito yonda* ‘read slowly’). Thus, if we specify a noun as a base (and a verb as a collocate), collocational units such as *hon-o yonda* ‘read a book’ can be extracted. If we specify a verb as a base (and a noun as a collocate), however, such collocational pairs cannot be fully detected since *yonda* ‘read’ potentially selects as an argument, e.g., *omosiroi hon* ‘interesting book,’ *tinpuna hon* ‘banal book,’ *kinô katta hon* ‘book which I bought yesterday,’ and so on.

Secondly, in extracting a sequence “noun + verb,” the choice of a noun or a verb as a base may affect results considerably. Consider (10).

**Table:** 

(10)	*gyôkan-o*	*yomu*	[Japanese]
	line.space-ACC	read	
	‘read between the lines’		

This expression consists of the verb *yomu* ‘read’ and the noun *gyôkan* ‘(actual) space between lines,’ but the meaning of the whole expression cannot be determined on the basis of these meanings encoded in each element. Although a lexical meaning of the verb *yomu* somehow persists in (10), it is not the case with the noun *gyôkan*. As will be argued in Section 3, a base in collocations must be “semantically transparent.” Thus, if we select a noun as a base, expressions such as (10) do not count as collocations.

Thirdly, a lexeme combination that is identified statistically as a collocation may turn out to be an idiom. Consider the following examples:

**Table:** 

(11)	*kûki-o*	*yomu*	[Japanese]
	air-ACC	read	
	‘act appropriately in context’

**Table:** 

(12)	*saba-o*	*yomu*	[Japanese]
	mackerel-ACC	read	
	‘provide disguised information (e.g. age)’		

In (11)–(12), neither the lexical meaning of a noun nor that of a verb persists. They are thus viewed as idioms, even if they may be statistically regarded as collocations. (See Section 3.6 for a more detailed discussion and illustration of the present issue.)

Finally, according to Table 1, nouns which denote something to read are strongly associated with the verb *yomu* ‘read.’ It is not quite obvious, however, whether these associations are cases of collocation or free combination in virtue of statistical results alone. We thus need the criteria for distinguishing collocations from free combinations.

Based on the above considerations, we specify a noun as a base in identifying a collocation “noun + verb.” In the next section, we will establish the Japanese-Korean criteria for extracting collocational units of the form “noun + verb,” to be distinguished from free combinations and idioms.

## 3 Hypothesis

The overall aim of this article is to offer the standard for collocations which may be employed for contrastive analyses of Japanese and Korean collocations. As stated at the outset, one of the empirical challenges encountered by the statistical analyses is how to identify collocations, to be separated from free combinations and idioms. This issue has largely been untouched for Japanese and Korean (Im, 2006; Lim, 2015; Yoo, 2012). Of special note is Im (2006), who provides the classification of clustered expressions in Korean and applies it to various collocational data (e.g. synonymous and antonymous paradigmatic relations in collocational clusters). In this section, we will demonstrate that Im’s (2006) criteria for Korean data (with slight amendments) are also applicable to Japanese. (Its empirical coverage will be further expanded in Section 4, where data that involve “Chinese-word” (kango, 漢語) will be analysed.)

### 3.1 Im (2006)

In Table 2, we present Im’s (2006: 174) classification of collocations, free combinations, and idioms (with slight amendments to be clarified shortly).

This classification is based on the two factors:

• Either a lexeme in a syntagmatic relation is replaceable with a synonymous lexeme or such replacement is (highly) constrained.
• A lexeme in a syntagmatic relation is semantically transparent or semi-transparent or non-transparent.

As displayed in Table 2, the former factor yields three types (A, B, C), and the latter factor yields five types (a, b, c, d, e). Each category is characterised by the combination of these two factors, as in “Aa,” which characterises free combinations. (These various combinations will be illustrated in due course; see Sections 3.2–3.4.)

In Table 2, the definitions of types “a, b, c, d, e” use the term “semi-transparent,” while Im (2006) employs the term “non-transparent” in place of “semi-transparent.” We contend that the term “semi-transparent” is more appropriate. Consider (13).

**Table:** 

(13)	*kelayl-ul*	*thuta*	[Korean]
	deal-ACC	open	
	‘enter into business relation’ (Im, 2006: 171)		

The original meaning of *thuta* is ‘make a way by removing a stacking thing.’ This meaning would be somehow related to the verbal part of ‘enter into business relation,’ and *thuta* is thus semantically “semi-transparent” in (13), which clearly contrasts with “non-transparent” cases like (14), where the original meaning of a consisting element is completely absent from the meaning of the whole part.

**Table:** 

(14)	*saba-o*	*yomu*	[Japanese]
	mackerel-ACC	read	
	‘provide disguised information (e.g. age)’		

In (14), the meaning of the whole expression ‘provide disguised information’ cannot be traced to the meanings of *saba* ‘mackerel’ and *yomu* ‘read’ in any sense. We thus use the term “non-transparent” for such cases as (14), and use the term “semi-transparent” for such cases as (13).

Im (2006) deals with only Korean data, but in the following subsections, we argue that Table 2 is also useful for capturing Japanese data.

### 3.2 Free Combinations

In Table 2, free combinations are characterised by the type “Aa.” That is to say, in a syntagmatic relation “α + β,” a co-occurring item β (verb) may be replaced with a synonymous lexeme. Furthermore, both α and β are semantically transparent.

**Table:** 

(15)	*kêi-o arawasu*	*yomu*	[Japanese]
	respect-ACC	show	
	‘show a respect (towards someone)’		

In (15), the verb *arawasu* ‘show’ is replaceable with other verbs such as *simesu* ‘show.’ Note also that the two consisting lexemes are semantically transparent; thus, *kêi* and *arawasu* here contribute their lexical meanings, ‘respect’ and ‘show’ respectively, to the overall meaning of the combined expression ‘show a respect.’

### 3.3 Collocations

Let us turn to collocations, which are divided into three subtypes: “Ba,” “Bb,” and “Cb.” These subtypes will be illustrated in turn.

In collocations of type Ba, consisting lexemes are both semantically transparent, as in the case of type Aa (Section 3.2). There are constrains, however, on the possibility of replacing a collocate (verb) with a synonymous lexeme.

**Table:** 

(16)	*bôsi-o*	*kaburu*	[Japanese]
	hat-ACC	put.on	
	‘put on a hat’

**Table:** 

(17)	*bôsi-o	*tukeru*	[Japanese]
	hat-ACC	attach	
	Int. ‘put on a hat’		

In (16), both *bôsi* ‘hat’ and *kaburu* ‘put on’ are semantically transparent. But the collocate *kaburu* cannot be replaced with *tukeru* ‘attach,’ as shown in (17), although they are similar in terms of lexical meaning in Japanese.

In type Bb, difficulty is also present in replacing a collocate with a synonym. But unlike type Ba, a collocate is semantically semi-transparent.

**Table:** 

(18)	*tyûmon-o*	*ukeru*	[Japanese]
	order-ACC	receive	
	‘take an order (at a restaurant)’		

The original meaning of *ukeru* is ‘receive’ but it means ‘take’ in (18), although some semantic similarity is still detected between ‘receive (an order)’ and ‘take (an order).’ In this sense, *ukeru* in (18) is semantically semi-transparent. In addition, *ukeru* cannot be replaced with the synonym *morau* ‘receive,’ as illustrated in (19).

**Table:** 

(19)	*tyûmon-o	*morau*	[Japanese]
	order-ACC	receive	
	Int. ‘take an order (at a restaurant)’		

In type Cb, a base is semantically transparent, whereas a collocate is semantically semi-transparent. Unlike type Bb, however, a collocate of type Cb is subject to heavier constraints on the replacement of a collocate with a synonymous lexeme. Type Cb, thus, blurs the distinction between collocations and idioms. (Examples of idioms will soon be given in the next subsection.) To pinpoint the problem, consider (20).

**Table:** 

(20)	*tosi-o*	*toru*	[Japanese]
	year-ACC	take	
	‘get old’		

In some dictionaries, (20) is registered as an idiom. This would be reasonable if we held that the base *tosi* ‘age’ were semantically non-transparent. As shown in (21), however, *tosi* also exhibits the meaning of ‘age.’

**Table:** 

(21)	*jussai-mo*	*tosi-ga*	*tigau*	[Japanese]
	10.year-even	age-NOM	different	
	Lit. ‘There is even a 10-year age difference.’			

Moreover, the collocate *toru* ‘take’ in (20) cannot be replaced with, say, *eru* ‘get.’

**Table:** 

(22)	*tosi-o	*eru*	[Japanese]
	year-ACC	take	
	Int. ‘get old’		

According to Table 2, therefore, (20) would be characterised as a collocation of type Cb, not a case of idioms.

### 3.4 Idioms

Let us finally examine idioms, which have three subcategories: “Cc,” “Cd,” and “Ce.”

As mentioned in the preceding subsections, in the cases of free combinations and collocations, a base is always semantically transparent. This is in sharp contrast with idioms, where a base is always not transparent (namely, always either “semi-transparent” or “non-transparent”). Another distinguished property of idioms is that the possibility of replacing a collocate with a synonymous lexeme is always highly restricted (or perhaps impossible).

In type Cc, although a noun is semantically semi-transparent, a collocate (verb) is transparent.

**Table:** 

(23)	*kuti-o*	*awaseru*	[Japanese]
	mouth-ACC	match	
	‘make their (inconsistent) stories look agree in front of the third persons’		

The noun *kuti*, which literally means a mouth, is used semi-transparently to denote a story, whereas the verb *awaseru* ‘match’ (or more precisely ‘make two things agree’) is semantically transparent.

In type Cd, consisting items are all semantically semi-transparent.

**Table:** 

(24)	*te-o*	*kiru*	[Japanese]
	hand-ACC	cut	
	‘break off the relationship with someone’		

In (24), *te* ‘hand’ means ‘relationship,’ and *kiru* ‘cut’ means ‘break off.’ Thus, though their lexical meanings are somehow related to the meaning of the whole expression (24), they are not identical, hence semantically semi-transparent.

Finally, type Cc represents the typical cases of idioms. Thus, in this category, the meaning of an idiomatic expression cannot, in any way, be related to the meanings of its composing elements. Example (25) is repeated from (14) in Section 3.1.

**Table:** 

(25)	*saba-o*	*yomu*	[Japanese]
	mackerel-ACC	read	
	‘provide disguised information (e.g. age)’		

The meaning of this idiomatic expression cannot be traced to the encoded meanings of the noun *saba* ‘mackerel’ and the verb *yomu* ‘read.’ Therefore, example (25) is a case of semantic non-transparency.

### 3.5 Ambiguous Cases

So far, the modified version of Im’s (2006: 174) criteria (Table 2) has been applied to Japanese data. Although this issue is neither noticed nor discussed in Im (2006), Table 2 can be applied to ambiguous cases. In Section 3.4, example (24) was presented as a case of idioms. This noun-verb pairing also possesses a free-combination reading.

**Table:** 

(26)		*te-o*	*kiru*	[Japanese]
		hand-ACC	cut	
	a.	‘break off the relationship with someone’		
	b.	‘cut a hand’		

The “a”-line specifies the idiomatic interpretation, and the “b”-line the free-combination interpretation. The standard in Table 2 is fully compatible with data like (26). First, (26) under the “a”-reading is of type Cd, a case of idiom. Second, the same string of words under the “b”-reading is of type Aa, a case of free combination (see Section 3.2).

Another example is provided in (27).

**Table:** 

(27)		*maku-o*	*aker-u*	[Japanese]
		curtain-ACC	open-DECL	
	a.	‘(a new thing) starts’		
	b.	‘open a curtain (e.g. in a theatre)’		

In the “a”-line, (27) would be of type Cd, a case of idiom. This “a”-reading is illustrated in (28). In the “b”-line, (27) is of type Aa, a case of free combination. This “b”-reading is illustrated in (29).

**Table:** 

(28)	*atarasii-jidai-ga*	*maku-o*	*ake-ta*	[Japanese]
	new-era-NOM	curtain-ACC	open-PAST	
	‘A new era has started.’

**Table:** 

(29)	*sihainin-ga*	*gekijô-no*	*maku-o*	*ake-ta*	[Japanese]
	manager-NOM	theatre-GEN	curtain-ACC	open-PAST	
	‘The manager opened the curtain of the theatre.’				

These ambiguous cases are naturally expected in our classification, since each type in Table 2 is independent from the other types. Im (2006), who only targets Korean data, does not consider ambiguous cases, but comparable data are found in Korean, too.

**Table:** 

(30)		*mwun-ul*	*tat-ta*	[Korean]
		door-ACC	close-DECL	
	a.	‘close the door’		
	b.	‘shut down, go out of business’		

In the “a”-line, (30) would be of type Aa, a case of free combination. This “a”-reading is illustrated in (31). In the “b”-line, (30) would be of type Cd, a case of idiom. This “b”-reading is illustrated in (32). ((31)–(32) were drawn from the novels included in the book entitled “푸른 수염의 첫 번째 아내” [The first wife of the guy with blue moustache], written by Seong-Ran Ha.)

**Table:** 

(31)	*chimsil-lo*	*tolawa*	*mwun-ul*	*tat-ass-ta*	[Korean]
	bedroom-into	return	door-ACC	close-PAST-DECL	
	‘He went back in the bedroom and closed the door.’

**Table:** 

(32)	*sayspyel*	*yuchiwen-un*	*mwun-ul*	*tat-ass-ta*	[Korean]
	S	nursery.school-TOP	door-ACC	close-PAST-DECL	
	‘The Sayspyel nursery school was closed up.’				

### 3.6 Summary

In this section, we have presented Im’s (2006) criteria with slight amendments and have demonstrated that it is applicable to not only Korean but also Japanese data.

As mentioned in Section 1, one of the challenges posed for statistical approaches to collocations is the empirical issue of whether it is reliable to identify collocational units purely in terms of statistical results. Consider (33)–(34).

**Table:** 

(33)	*katudô-o*	*suru*	[Japanese]
	activity-ACC	do	
	‘act’

**Table:** 

(34)	*hara-o*	*tateru*	[Japanese]
	stomach-ACC	evoke	
	‘get angry’		

These syntagmatic relations (33)–(34) would statistically count as collocations. Consider Table 3 and Table 4 below.

According to Hunston (2002), a syntagmatic relation may be statistically construed as a collocation if *Freq.* is on or more than 10, each of *fA* and *fB* is on or more than 100, the *MI*-score is on or more than 3.0, and the *t*-score is on or more than 2.0. As for (33), the *MI*-score and *t*-score in Table 3 mark high values (in particular, the *t*-score); it is then suggested that *katudô-o suru* constitutes a collocational unit that is frequently used. As for (34), consider Table 4. The Dice Coefficient, which calculates the collocational strength based on the frequency relation between *hara* ‘stomach’ and *tateru* ‘evoke,’ marks a high value. Furthermore, the *MI*-score and the *t*-score are also high. It is thus suggested that *hara-o tateru* constitutes a collocational unit. (For comparison purposes, Table 4 presents other data involving *hara* ‘stomach’: *hara-o kukuru* ‘make up one’s mind.’ As in (34), the Dice Coefficient, the *MI*-score, and the *t*-score for this sequence are high, and it would be statistically treated as a collocation.)

However, with respect to our criteria introduced in this section (Table 2), (33)–(34) are not viewed as collocations. In (33), *katudô* ‘activity’ and *suru* ‘do’ are semantically transparent, and this sequence of words is characterised as type Aa, an instance of free combinations. In (34), the contribution of *hara* ‘stomach’ to the overall meaning of the sequence is semi-transparent. On the other hand, *tateru* is polysemous and it exhibits the meaning of ‘evoke (an emotion)’ in this example; *tateru* is thus semantically transparent. With respect to Table 2, then, (34) is classified as type Cc, a case of idioms. (Further, the semantic contributions of *hara* ‘stomach’ and *kukuru* ‘tie up’ to the overall meaning of *hara-o kukuru* ‘make up one’s mind’ are not transparent. Thus, according to Table 2, it is also regarded as an idiom of type Cd.)

To sum up, the identification of collocations is not entirely achieved if statistical results alone are taken into account, and it is important to establish the standard against which collocations in Japanese and Korean are appropriately characterised and are also properly distinguished from free combinations and idioms. In the next section, we will further argue that our criteria are also useful for identifying collocational sequences that involve “Chinese-words” (kango, 漢語) in the two languages.

## 4 Analysis

The last section has presented the Japanese-Korean criteria for identifying collocations by making slight modifications to Im’s (2006) proposal. In the present section, we will further confirm the usefulness of the criteria for Japanese and Korean by exploring the syntagmatic units which contain expressions mutually observed in the two languages: “Chinese-words” (kango, 漢語).

Chinese-words account for the large proportion of the vocabulary in Japanese and Korean. (Yamaguchi et al. (2004: 115) reports that Chinese-words amount to 45.89% of the Japanese vocabulary.) They thus serve as a reasonable starting point for providing a Japanese-Korean contrastive analysis. Furthermore, Chinese-words differ from the other types of word such as “wago” (Japanese-native words) and loanwords in that a number of Chinese-words denote an action and they are often combined with the light verbs (Muraki, 1991: 203): *suru* ‘do’ in Japanese (35) and *hata* ‘do’ in Korean (36). (It may then be expected that Chinese-words denoting an action and those denoting a non-action (e.g. state) co-occur with different types of verb. The present article investigates only action-denoting Chinese-words, providing a partial basis for comparison in future work.)

**Table:** 

(35)	*kôdô-suru*	[Japanese]
	behaviour-do	
	‘behave’

**Table:** 

(36)	*hayngtond-hata*	[Korean]
	behaviour-do	
	‘behave’	

We concentrate on the syntagmatic relation “Chinese-word + case particle + verb.” In this sequential pattern, a Chinese-word (together with a case particle) is treated as a base, whereas a verb is treated as a collocate.

The lists of Chinese-words used in our survey are listed in (37) (for Japanese) and in (38) (for Korean).

**Table:** 

(37)	Chinese-words: Japanese
	*kôdô* (行動) ‘behaviour,’ *kyôkan* (共感) ‘sympathy,’ *tyûmoku* (注目) ‘attention,’ *kensa* (検査) ‘inspection’ *tyôsa* (調査) ‘enquiry,’ *henka* (変化) ‘change,’ *kyôkyû* (供給) ‘supply,’ *kandô* (感動) ‘moving,’ *keikaku* (計画) ‘plan,’ *hanketu* (判決) ‘judge’

**Table:** 

(38)	Chinese-words: Korean
	*hayngtong* (行動) ‘behaviour,’ *kongkam* (共感) ‘sympathy,’ *cwumok* (注目) ‘attention,’ *kemsa* (檢査) ‘inspection’ *cosa* (調査) ‘enquiry,’ *pyenhwa* (變化) ‘change,’ *kongkup* (供給) ‘supply,’ *kamtong* (感動) ‘moving,’ *kyeyhoyk* (計劃) ‘plan,’ *phankyel* (判決) ‘judge’

For statistical types of research, it may not be quantitatively sufficient to target only these Chinese-words with the specified schema “Chinese-word + case particle + verb.” In this article, however, we are not engaged in presenting a rigid statistical account, but attempt to (i) point out empirical problems for extracting collocations in Japanese and Korean, (ii) set out the standard for identifying collocations in these languages, and (iii) test this standard against specific data involving Chinese-words. A more large-scale exploration is thus left for future research.

Next, the lists of case particles exploited are presented in (39) (for Japanese) and in (40) (for Korean).

**Table:** 

(39)	Case particles: Japanese
	*ga* (nominative), *o* (accusative), *ni* (dative), *de* (locative), *e* (allative), *kara* (ablative)

**Table:** 

(40)	Case particles: Korean
	*i*/*ka* (nominative), (*l*)*ul* (accusative), *ey* (dative), (*u*)*lo* (allative), *eyse* (locative, ablative)

We first calculate the co-occurrence frequencies of the Chinese-words and a case particle in our corpora. The result is summarised in Table 5 (For reasons of space, only the *t*-scores are provided here).

Except for the Chinese-word meaning ‘supply’ in Korean, the other Chinese-words are most frequently marked with the accusative case particle: *o* in Japanese and (*l*)*ul* in Korean. Akimoto (1993) reports similar results for the case-marking of Chinese-words in Japanese. As for the Chinese-word for ‘supply’ in Korean, it is marked with the nominative particle *i*/*ka* or the accusative particle (*l*)*ul* to a similar degree. Given these results, we restrict our attention to the combinations of the Chinese-words with the accusative case particles (*o* in Japanese and (*l*)*ul* in Korean).

The sequences “Chinese-word + accusative case particle” being specified, we then identify collocations among them by assuming that a Chinese-word is a base and a verb is a collocate. This process relies on the criteria for collocations introduced in Table 2. The result is shown in Table 6. (Different notations for a lexeme count as one and the same lexeme. For instance, *okonau* ‘do’ may be expressed as “行う” or “行なう,” but these are treated as different manifestations of the single lexeme *okonau*.) Of special note is that a much wider range of verbs is allowed as a collocate in Japanese, as will be discussed below.

First, the set of verbs (as collocates) in Korean forms a proper subset of the set of verbs in Japanese for eight Chinese-words meaning ‘behaviour,’ ‘sympathy,’ ‘attention,’ ‘inspection’ ‘supply,’ ‘moving,’ ‘plan,’ and ‘judge,’ respectively. In particular, the following Chinese-words in Korean co-occur with only a couple of verbs: *hayngtong* ‘behaviour,’ *kongkam* ‘sympathy,’ *cwumok* ‘attention,’ *kemsa* ‘inspection,’ *kongkup* ‘supply,’ *kamtong* ‘moving,’ and *phankyel* ‘judgement.’ Further, free combinations are included in this list, such as *hayngtong hata* (Lit. ‘do behaviour’), where *hata* is a light verb ‘do.’ Moreover, the verb *patta* ‘receive,’ co-occurring with *kongkup* ‘supply’ and *kamtong* ‘moving,’ is not marked with a case particle, and they do not live up to our standard for collocations.

Note that the small frequency of a noun does not necessarily indicate that the variation of co-occurring verbs is also small. For instance, consider the Chinese-word *dentatu* ‘transmission.’ The *fA* of *dentatu* is 74, but it co-occurs with a wide range of verbs, as illustrated in (41)–(43).

**Table:** 

(41)	*dentatu-o*	*suru*	[Japanese]
	transmission-ACC	do	
	‘transmit’

**Table:** 

(42)	*dentatu-o*	*okonau*	[Japanese]
	transmission-ACC	conduct	
	‘transmit’

**Table:** 

(43)	*dentatu-o*	*hakaru*	[Japanese]
	transmission-ACC	intend	
	‘intend to transmit’		

In these (and other) combinations involving *dentatu*, the *MI*-score is more than 3.0, though the *t*-score for (42) and that for (43) are below 2.0 due to the small frequency of the Chinese-word (see Section 3.6). When the frequency of a Chinese-word is small, the type of combination (i.e. free combination, collocation, idiom) can be identified based on our criteria presented in Table 2. For instance, *okonau* in (42) can be replaced as *suru* (see (41)), and *dentatu-o okonau* is thus classified as a free combination. In this way, the present article raises the empirical issues for the (purely) statistical treatments of collocations, and provides the means of identifying collocations in terms of lexeme-replacement and semantic transparency.

Second, collocations with ‘change’ vary across the two languages. Whilst Japanese exhibits 22 patterns, Korean exhibits only 14 patterns. Notably, although collocational patterns in Korean generally do not display much variation, the Chinese-word meaning ‘change’ in Korean co-occurs with several verbs which are absent from the list of verbs allowed in Japanese. The relevant examples include *pyenhwa-lul kyekkta* ‘experience a change,’ *pyenhwa-lul ikkulta* ‘attract experience,’ *pyenhwa-lul kkoyhata* ‘attempt a change,’ and so forth.

Finally, a discrepancy lies in the Chinese-words for ‘enquiry’ in the two languages. In Japanese, only the verb *okonau* ‘conduct’ is identified as a collocate of this Chinese-word. In Korean, *pel’ita* ‘make it happen’ is identified as a collocate; the corresponding Japanese verb never co-occurs with the Chinese-word meaning ‘enquiry.’

Overall, it is indicated that whilst some idiosyncratic differences can be observed, a much wider variety of co-occurring verbs are found in Japanese than in Korean.

## 5 Conclusion

The study of collocations raises various empirical problems. In particular, in order to extract collocations from corpora, it is important to establish the criteria for determining collocations (to be distinguished from free combinations and idioms). In this article, we have concentrated on these empirical issues (rather than rigid statistical analyses), and have shown that the criteria proposed for Korean collocations by Im (2006), with slight modifications, is extendable to Japanese in a uniform way. Based on these criteria, we have presented a case study of the syntagmatic sequence “Chinese-word + case particle + verb” in the two languages, hoping that our methodological/empirical discussions will be fruitfully combined with statistical approaches to collocations. One of our future prospects is to refine the criteria for collocations by addressing other aspects of the phenomena than “substitution of composing units” and “semantic transparency” (cf. Im (2006)) and by covering a wider spectrum of data within/beyond the “Chinese-word + case particle + verb” pattern. Further work in this direction, we hope, will shed light on lexical and grammatical facets of Japanese and Korean, and it will also contribute to the building of a Japanese-Korean collocation database for contrastive linguistic research.

## Declarations

### Author contribution statement

Jong Seung Park, Tohru Seraku: Conceived and designed the experiments; Analyzed and interpreted the data; Wrote the paper.

Jieun Kiaer: Conceived and designed the experiments; Analyzed and interpreted the data.

### Competing interest statement

The authors declare no conflict of interest.

### Funding statement

Jong Seung Park’s part of this work was supported by the National Research Foundation of Korea Grant funded by the Korean Government (NRF-2013S1A2A1A01034308). Tohru Seraku’s part of this work was supported by Hankuk University of Foreign Studies Research Fund of 2016.

### Additional information

No additional information is available for this paper.

## Figures and Tables

**Table 1:** The Verb *Yomu* ‘read’ in Japanese and its Co-occurring Nouns.

base	*Freq.*	*fA*	*fB*	*w*	*D*	*MI*	*T*
*hon* ‘book’	3356	26502	75755	2530000000	0.07	12.05	57.92
*kiji* ‘article’	1280	26502	47775	2530000000	0.03	11.32	35.76
*sinbun* ‘newspaper’	432	26502	26502	2530000000	0.02	10.60	20.77
*tyôji* ‘memorial address’	9	26502	54	2530000000	0.00	13.96	3.00
*tyûigaki* ‘notice’	37	26502	279	2530000000	0.00	13.63	6.08
*gyôkan* ‘space between lines’	22	26502	180	2530000000	0.00	13.51	4.69
*saba* ‘mackerel’	13	26502	334	2530000000	0.00	11.86	3.60
*k*û*ki* ‘air’	55	26502	6960	2530000000	0.00	9.56	7.41

**Table 2:** The Criteria for Collocations (Im, 2006: 174, revised).

Free combinations	Collocations			Idioms		
A	B	B	C	C	C	C
a	a	b	b	c	d	e

A. lexeme-replacement is free.

B. lexeme-replacement is constrained.

C. lexeme-replacement is highly constrained.

a. transparent + transparent.

b. transparent + semi-transparent.

c. semi-transparent + transparent.

d. semi-transparent + semi-transparent.

e. non-transparent.

**Table 3:** The statistical scores for (33).

*Freq.*	*fA*	*fB*	*w*	*MI*	*T*
1290	7259	2919140	253000000	3.95	33.58

**Table 4:** The statistical scores for (34).

	*Freq.*	*fA*	*fB*	*w*	*D*	*MI*	*T*
*tateru*	336	974	7340	253000000	0.08	13.54	18.33
*kukuru*	71	974	642	253000000	0.09	14.81	8.43

**Table 5:** The Co-occurrence Frequencies for “Chinese-word + Case Particle”.

	Japanese						Korean				
	*ga*	*o*	*ni*	*de*	*e*	*kara*	*i/ka*	*ul/lul*	*ey*	*(u)lo*	*eyse*
‘behaviour’	29.71	58.92	38.56	10.48	7.17		18.83	32.07	18.28	16.01	3.99
‘sympathy’	4.93	19.88					5.57	13.92			
‘attention’	7.58	32.86						16.24			
‘inspection’	12.63	33.32	13.68	15.82			7.22	18.06	4.97	3.50	5.27
‘enquiry’		47.45	33.87	34.86		6.83	14.25	33.28	17.60	6.13	17.07
‘change’	40.12	42.40	38.67		9.09		30.81	39.12	26.74	11.37	4.21
‘supply’	14.06	15.94	5.85		1.85		11.56	10.65	5.22	3.57	0.81
‘moving’	11.21	28.41					8.06	19.34	5.45	6.39	
‘plan’	24.30	53.05	32.78	18.33	4.91		19.82	33.66	18.27	8.17	4.20
‘judgement’	24.35	29.87	16.02	12.59	2.14		11.99	16.68	8.49	5.01	6.68

**Table 6:** The List of Collocations “Chinese-word + Case particle + Verb”.

Base	Collocate			Japanese				Korean			
	Japanese	meaning	Korean	Freq.	D	MI	T	Freq.	D	MI	T
*kôdô* (J) *hayngtond* (K) ‘behaviour’	*toru*	‘take’	*chwihata*	696	0.02	9.04	26.33	81	0.02	10.79	8.99
	*okosu*	‘initiate’		524	0.08	11.78	22.88				
	*suru*	‘do’	*hata*	392	0.00	3.10	17.49	455	0.00	6.41	21.08
	*okonau*	‘conduct’		80	0.00	5.87	8.79				
	*kimeru*	‘determine’		20	0.00	6.23	4.41				
	*toreru*	‘can take’		18	0.00	6.97	4.21				
	*simesu*	‘suggest’		18	0.00	5.77	4.17				
	*okoseru*	‘can initiate’		17	0.01	12.85	4.12				
	*tomeru*	‘stop’		14	0.00	6.84	3.71				
	*miseru*	‘show’	*poita*	12	0.00	5.81	3.40	81	0.01	8.43	8.97
	*sasaeru*	‘stand’		10	0.00	6.58	3.13				
	*motomeru*	‘demand’		10	0.00	4.78	3.05				
	*rissuru*	‘regulate’		6	0.00	11.48	2.45				
*kyôkan* (J) *kongkam* (K) ‘sympathy’	*oboeru*	‘remember’		93	0.01	9.46	9.63				
	*yobu*	‘call’	*pwuluta*	72	0.01	8.18	8.46	7	0.00	7.58	2.22
	*idaku*	‘embrace’		10	0.00	7.62	3.15				
	*eru*	‘get’	*etta*	68	0.00	7.53	8.20	45	0.01	11.12	6.71
	*simesu*	‘suggest’		15	0.00	6.16	3.82				
	*ataeru*	‘give’		14	0.00	5.88	3.68				
	*motu*	‘hold’		33	0.00	5.46	5.61				
*tyûmoku* (J) *cwumok* (K) ‘attention’	*atumeru*	‘put together’		860	0.16	14.13	29.32				
	*abiru*	‘bask’		312	0.16	14.44	17.66				
	*hiku*	‘draw’	*kkulta*	18	0.00	8.43	4.23	92	0.03	11.44	9.59
	*eru*	‘get’		13	0.00	6.07	3.55				
	*ukeru*	‘receive’	*patta*					176	0.01	9.42	13.25
*kensa* (J) *kemsa* (K) ‘inspection’	*ukeru*	‘receive’	*patta*	318	0.01	10.47	17.82	111	0.01	9.43	10.52
	*okonau*	‘conduct’		167	0.00	8.56	12.89				
	*yaru*	‘do’		19	0.00	5.31	4.25				
	*kobamu*	‘reject’		6	0.01	10.66	2.45				
	*yattemiru*	‘try doing’	*haypota*					7	0.01	11.26	2.64
*tyôsa* (J) *cosa* (K) ‘enquiry’	*okonau*	‘conduct’	*haynghata*	686	0.02	9.42	26.15	7	0.00	7.22	2.63
	*susumeru*	‘put forward’		98	0.01	8.81	9.88				
	*tuzukeru*	‘continue’		45	0.00	7.02	6.66				
	*yaru*	‘do’		37	0.00	5.12	5.91				
	*ukeru*	‘receive’		31	0.00	6.02	5.48	191	0.01	7.61	13.75
	*kasaneru*	‘pile up’		11	0.00	8.08	3.30				
	*okosu/hassu*	‘produce’	*pel’ita*					140	0.03	10.06	11.82
	*heru*	‘pass’	*kechita*					12	0.00	7.04	3.44
	*hikiukeru*	‘undertake’	*mathta*					8	0.00	6.22	2.79
	*yattemiru*	‘try doing’	*haypota*					7	0.00	7.22	2.63
*henka* (J) *pyenhwa* (K) ‘change’	*motarasu*	‘bring’	*kacyeota*	148	0.04	11.43	12.16	103	0.02	10.35	10.14
	*togeru*	‘undergo’	*ilwuta*	89	0.05	12.94	9.80	8	0.00	4.86	2.73
	*okosu*	‘initiate’	*il’ukhita*	69	0.01	9.95	8.30	71	0.01	8.94	8.41
	*tukeru*	‘attach’		75	0.00	7.83	8.62				
	*miru*	‘see’	*slphyepota*	76	0.00	5.08	8.46	12	0.00	6.78	3.43
	*ataeru*	‘give’	*cwuta*	41	0.00	7.60	6.37	56	0.01	6.66	7.41
	*motomeru*	‘demand’	*chacta*	32	0.00	7.29	5.62	11	0.00	4.59	3.18
	*simesu*	‘suggest’		29	0.00	7.29	5.35				
	*miseru*	‘show’		28	0.00	7.85	5.27				
	*tanosimu*	‘enjoy’		23	0.00	7.65	4.77				
	*tuzukeru*	‘continue’		23	0.00	6.43	4.74				
	*tomonau*	‘accompany’		19	0.00	8.33	4.35				
	*humaeru*	‘consider’		18	0.01	9.31	4.24				
	*ukeru*	‘receive’		15	0.00	5.35	3.78				
	*unagasu*	‘accelerate’		14	0.01	9.45	3.74				
	*motu*	‘hold’		15	0.00	4.49	3.70				
	*hikiokosu*	‘bring about’		13	0.01	9.31	3.60				
	*ukeireru*	‘accept’	*patatulita*	13	0.00	7.75	3.59	5	0.00	5.23	2.18
	*toraeru*	‘capture’		17	0.00	8.28	4.11				
	*yomu*	‘read’	*ilkta*	12	0.00	4.38	3.30	10	0.00	5.00	3.06
	*oyobosu*	‘exert’		10	0.00	8.82	3.16				
	*kawaeru*	‘add’	*kahata*	10	0.00	6.62	3.13	5	0.00	6.83	2.22
	*keikensuru*	‘experience’	*kyekkta*					76	0.01	9.29	8.70
	*hipparu*	‘pull’	*ikkulta*					13	0.00	7.28	3.58
	*hakaru*	‘intend’	*kkoyhata*					11	0.00	9.53	3.31
	*heru*	‘pass’	*kechita*					7	0.00	7.25	2.63
	*konomu*	‘favour’	*cohahata*					5	0.00	5.17	2.17
*kyôkyû* (J) *kongkup* (K) ‘supply’	*okonau*	‘conduct’		42	0.00	4.73	6.24				
	*ukeru*	‘receive’	*patta	37	0.00	5.44	5.94	64	0.00	8.15	7.97
	*huyasu*	‘increase’	*nullita*	14	0.00	7.10	3.71	10	0.01	10.19	3.16
*kandô* (J) *kamdong* (K) ‘moving’	*ataeru*	‘give’	*cwuta*	184	0.01	10.90	13.56	129	0.02	10.30	11.35
	*oboeru*	‘memorise’		164	0.02	11.57	12.80				
	*ajiwau*	‘taste’		39	0.01	10.96	6.24				
	*yobu*	‘call’		38	0.00	8.59	6.15				
	*tutaeru*	‘tell’		23	0.00	8.53	4.78				
	*eru*	‘get’		18	0.00	6.95	4.21				
	*umu*	‘produce’		16	0.01	10.33	4.00				
	*ukeru*	‘receive’	*patta	15	0.00	6.51	3.83	60	0.00	8.14	7.72
	*yobiokosu*	‘evoke’		14	0.02	13.41	3.74				
	*sasou*	‘invite’		11	0.00	9.30	3.31				
	*huyasu*	‘increase’	*nullita*					8	0.01	9.94	2.83
*keikaku* (J) *kyeyhoyk* (K) ‘plan’	*tateru*	‘set up’	*seywuta*	730	0.12	12.65	27.01	319	0.08	12.71	17.86
	*neru*	‘knead’	*ccata*	91	0.03	10.93	9.53	30	0.01	11.37	5.48
	*susumeru*	‘put forward’		80	0.01	8.81	8.92				
	*motu*	‘hold’	*kacta*	77	0.00	5.93	8.63	36	0.00	6.55	5.94
	*tukuru*	‘make’		102	0.00	6.91	10.02				
	*sadameru*	‘determine’		41	0.01	9.05	6.39				
	*kangaeru*	‘think’		30	0.00	4.09	5.16				
	*minaosu*	‘review’		21	0.00	8.14	4.57				
	*okonau*	‘conduct’		22	0.00	4.43	4.47				
	*utidasu*	‘type out’	*naynohta*	20	0.01	10.19	4.47	9	0.00	6.91	2.98
	*dasu*	‘submit’		18	0.00	5.01	4.11				
	*simesu*	‘suggest’		14	0.00	5.65	3.67				
	*matomeru*	‘summarise’		13	0.00	6.45	3.56				
	*kimeru*	‘decide’	*capta*	12	0.00	5.73	3.40	10	0.00	4.88	3.05
	*hukumu*	‘include’		11	0.00	5.45	3.24				
	*torimatomeru*	‘put together’		6	0.00	10.27	2.45				
	*akasu*	‘uncover’	*palkhita*					103	0.01	8.19	10.11
	*maedaosinisuru*	‘accelerate’	*aphtangkita*					6	0.00	9.22	2.45
*hanketu* (J) *phankyel* (K) ‘judgement’	*ukeru*	‘receive’	*patta*	183	0.01	10.05	13.52	68	0.02	10.60	8.24
	*iiwatasu*	‘sentence’		159	0.20	16.02	12.61				
	*kudasu*	‘pronounce’	*naylita*	139	0.10	14.27	11.79	133	0.03	11.46	11.53
	*dasu*	‘output’		60	0.00	8.48	7.72				
	*motomeru*	‘demand’		24	0.00	8.01	4.88				
	*eru*	‘get’	*etta*	10	0.00	6.08	3.12	5	0.00	6.87	2.22
	*kudasareru*	‘be pronounced’		8	0.01	12.36	2.83				
	*katitoru*	‘win’		7	0.01	11.68	2.64				
	*kutugaesu*	‘overturn’		7	0.01	11.46	2.64				
